# Infection Prevention Control (IPC) and Antimicrobial Resistance (AMR)

**DOI:** 10.1007/978-3-030-62662-4_4

**Published:** 2020-11-24

**Authors:** Louise Ackers, Gavin Ackers-Johnson, Joanne Welsh, Daniel Kibombo, Samuel Opio

**Affiliations:** 6 Global Social Justice, University of Salford, Salford, UK; 7 University of Salford, Salford, UK; 8 Infectious Disease Institute, Kampala, Uganda; 9 Pharmaceutical Society of Uganda, Kampala, Uganda

**Keywords:** Infection Prevention Control, Maternal sepsis, Wound management, Surgical Site Infection, Health Care Acquired Infection, Hand hygiene

## Abstract

This chapter outlines a key component of improved AMR; namely infection prevention control (IPC). It addresses some of the issues most commonly associated with IPC including hand hygiene, waste disposal and infrastructure. It then addresses wound management as an Infection Control issue. The emergence of wound management as a central focus in the Maternal Sepsis Intervention proved pivotal in shaping the pathway to antimicrobial stewardship.

## ‘Prevention First’

In 2017, the WHO adopted a Resolution focused on improving the prevention, diagnosis, and management of sepsis. Reinhart et al. underline the importance of recognising sepsis as a global health priority. They go on to suggest that progress towards ‘a world free of sepsis’ requires recognition of the key role of prevention (2017: 416). Infection prevention reduces the overuse of antibiotics which drives resistance. Infection-Prevention-Control (or ‘IPC’) has featured strongly on the global health agenda for many years with significant emphasis on those infections that patients (and health workers) acquire within health facilities. Whilst attempts to tackle the source of infection in the home and workplace have formed the basis of HIV-awareness and public vaccination programmes, the phenomena known as ‘Health Care Acquired Infection’ (HCAIs) and its cousin, ‘ Surgical Site Infection’ (SSIs) have focussed concern on ‘adverse’ events associated with hospitalisation.

## What Is a Health Care Acquired Infection?

The World Health Organisation’s Patient Safety Fact File (2019) defines ‘Health Care Associated Infection’ as follows:

Health care-associated infections, or “nosocomial” and “hospital” infections, affect patients in a hospital or other health-care facility, and are not present or incubating at the time of admission. They also include infections acquired by patients in the hospital or facility but appearing after discharge, and occupational infections among staff.^1^

The Fact File reports health care associated infection rates of 10% amongst hospitalised patients in LMICs (2019: 9). Allegranzi et al.’s systematic review of health-care-associated infection in developing countries found that the prevalence of health-care-associated infection is ‘much higher’ in LMICs than HICs and concluded that Surgical Site infection was the ‘leading health care-associated infection in the developing world’ (2011: 28).

## What Is a Surgical Site Infection?

The Centre for Disease Control (CDC) defines a Surgical Site Infection (SSI) as, ‘an infection that occurs after surgery in the part of the body where the surgery took place’ and occurring within 30 days after the procedure (or 12 months in the case of orthopaedic implants).^2^ Seni et al.’s (2013) analysis of 314 SSI cases at Uganda’s national referral hospital reported a much higher incidence of SSIs amongst women (76.1% of their sample) and a preponderance of cases in obstetrics and gynaecology wards (62.1%). Caesarean-section and laparotomy^3^ accounted for more than three quarters of all surgical procedures in their study. They conclude:

The predominance of SSIs in obstetrics and gynaecology wards is quite alarming and thus, a need to institute stringent infection prevention and control measures in this setting, more especially in emergency surgeries which accounted for more SSIs cases as opposed to elective surgeries. (2013: 5)

A similar study in Tanzania (Mawalla et al. 2011) reported SSI rates of 26% amongst patients undergoing surgery and similarly noted the gendered impact of SSIs largely reflecting the volume of women having surgery in the first place.^4^

Following the award for the MSI, the funding bodies added an additional ‘request’ that each team conduct a Global Point Prevalence Survey (G-PPS).^5^ This is a standardised survey of antimicrobial use amongst all in-patients in a hospital on a given day designed to deliver comparative data for international benchmarking. It captures that data through documentation in patient records (which may be an inaccurate reflection of practice). The MSI team undertook the GPPS on May 7th, 2019. It involved all 42 patients on the post-natal and gynae wards at 8 am (22 in gynae and 20 in post-natal). The GPPS found that 94% patients on post-natal ward were prescribed antibiotics for surgical prophylaxis (94%) with one case involving suspected Community Acquired Infection (CAI).^6^ The picture in the adjoining gynae ward was quite different. Here, 45% patients were prescribed antibiotics and 50% of these were related to a suspected health care associated Infection (Table 4.1).

**Table 4.1:** Antibiotic Prescribing on Post-natal and Gynaecology Wards in FPRRH (GPPS)

	Gynaecology	Post-natal
Total no of patients	22	20
Percentage of patients on antibiotics	45	90
Percentage of antibiotics for Community acquired infection	20	6
Percentage of antibiotics for Health-care-associated infection	50	0
Percentage of antibiotics for medical prophylaxis	0	0
Percentage of antibiotics for surgical prophylaxis	10	94
Percentage of antibiotics for unknown indication	20	0

*Source* Results of G-PPS, May 2019 as reported to FPRRH IPC Committee

## Infection Prevention and Control in the Ugandan National Action Plan

**Fig. 4.1:**
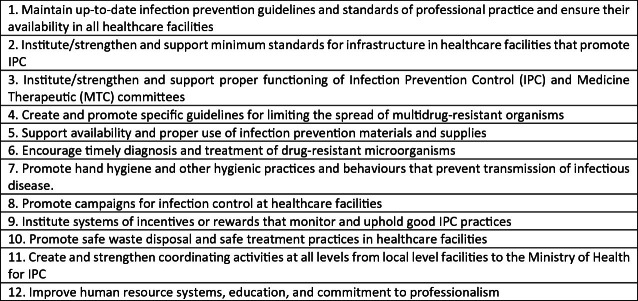
Objective 3.2.1 Strengthen Infection Prevention and Control Programmes (*Source* Ugandan National Action Plan on AMR)

The Ugandan National Action Plan on AMR outlines its ‘ One Health’ approach arguing that, ‘Prevention is the most effective, affordable way to reduce risk for and severity of resistant infections’ (2019: 6). Strategic Objective Two identifies key actions to improve infection prevention and control. These span four inter-linked areas: IPC in healthcare facilities; IPC in the community; biosecurity in agriculture and vaccination programmes. Action 3.2.1 sets out key objectives to ‘Strengthen Infection Prevention and Control Programs in Healthcare Facilities’ and is the area of most direct relevance to the Maternal Sepsis Intervention (Fig. 4.1).

Objective 3.2.1 reiterates well-rehearsed (if neglected) IPC goals: to raise awareness; improve hand hygiene, basic infrastructure, and waste disposal. Goals 4 and 6 add a specific AMR ‘twist’ and illustrate the immediate connection with the surveillance objectives outlined in Strategic Objective 4. The creation of guidelines to limit the spread of multidrug-resistant organisms and timely diagnosis and treatment of drug-resistant organisms requires strong multi-disciplinary team working with laboratory scientists, pharmacists, doctors, nurses and midwives.

This chapter reports first on the more familiar aspects of IPC concerned primarily with creating an environment on the post-natal and gynaecology wards that reduces opportunities for women entering that ward to acquire a health care-acquired infection as a direct result of practices on that ward. The ‘control’ component of IPC is often neglected; IPC is not just about prevention; it is also about controlling infection. Many women arriving on the PNG will already have been exposed to risks of HCAI and SSI either in the operating theatres and labour wards at the same hospital or in the referring facilities they pass through on their journey into the hospital. In such cases, the focus on the PNG is on early identification of infection and appropriate management. Wound management has emerged as a key concern in the control of infection for those women with infected wounds; for the women and attendants around them and for the health workers caring for them as they become a source of infection to others.

## IPC in the Maternal Sepsis Intervention^7^

The project team were aware of the central importance of IPC to antimicrobial resistance when we applied for funding. Arguably the emphasis in the literature on AMR has focused too much on the management of antibiotics by individual health workers and patients which, to use a colloquial expression from the UK, amounts to, ‘locking the door after the horse has bolted’. Denyer-Willis and Chandler emphasise the importance of preventive approaches:

Antibiotics have become …. a quick fix for hygiene in settings of minimised resources. (2019: 1)

This is particularly relevant in LMICs, where, they argue, antimicrobials are, ‘*put to work to correct the fractured infrastructures of care, water and sewage, hygiene and demands for ever increasing [health worker] productivity*’ (p. 2).

We have seen this in previous K4C work on antibiotic stewardship. Women at a health centre III were being routinely prescribed prophylactic antibiotics following vaginal birth as a mechanism to protect against uncertainty surrounding hygiene and sanitation in both the hospital setting and the home setting (Welsh 2019).

Denyer-Willis and Chandler (2019) emphasise the importance of ‘connectivities’ and underline the need for multi-disciplinary teams and methods (including social science and anthropological approaches) in order to present an holistic and accurate picture of the deeply contextual factors contributing to AMR and potential responses. Prevention must be the starting point of all holistic AMR interventions; it is also the most cost-effective.

##  Hand Hygiene

Maina et al. describe **wa**ter, **s**anitation, and **h**ygiene (WASH) as the key foundations of AMR in Kenyan hospitals:

Poor WASH increases hospital-associated infections and contributes to the rise of antimicrobial resistance. (2019: 1)

The survey tool developed by Maina et al. and piloted in 14 general hospitals in Kenya showed major performance variations between hospitals and wards reflecting differences in the built environment, resource availability and leadership. They identify waste management and (healthworker) hand hygiene as ‘critical indicators’ with hand hygiene achieving an aggregate score across all facilities of only 35% (p. 1). Allegranzi et al. report even lower levels of hand hygiene compliance of around 20% in LMICs (2011: 235). Hand hygiene compliance at FPRRH was observed to sit at a mere 17.4% in 2018 (Mbabazi 2018).

**Fig. 4.2:**
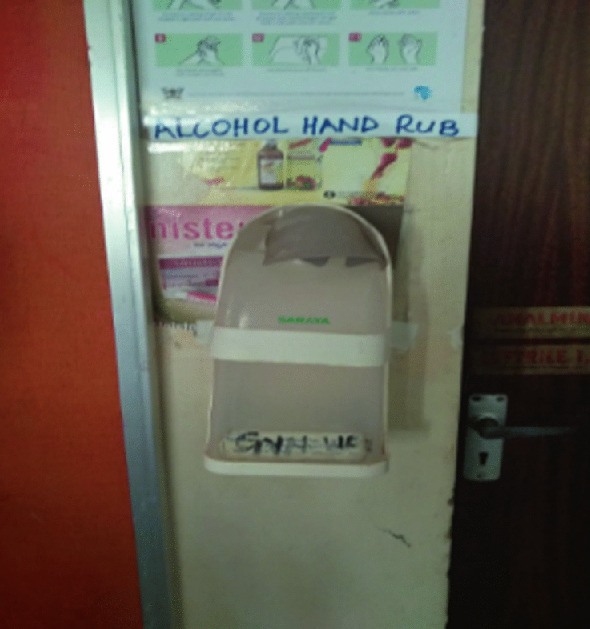
Empty and damaged hand gel dispenser next to Ugandan guidelines on handwashing

K4C previously held a grant (awarded in 2015) from the Tropical Health and Education Trust which focused on IPC in the Kabarole region. In keeping with our experience of behaviour change in Uganda, the hand hygiene project combined formal training with continuous mentoring whilst commencing quality controlled local manufacture of alcohol-based hand sanitiser. Providing training without ensuring that health workers had access to the opportunity to exercise that knowledge was, we felt, arrogant and insulting. The hand hygiene project achieved a considerable shift in health worker behaviour; but only for as long as K4C was in a position to fund the costs of the hand gel. Despite persuasion, facilities proved unwilling to contribute in any way at all to the constituent (and cheap) ingredients for hand gel production. When the MSI commenced, we immediately expected and noticed the absence of hand gel. The juxtaposition of a broken and empty hand gel dispenser next to the Ugandan guidelines on hand hygiene on entry to the ward can only have had a demotivating impact on health worker behaviour especially when dealing with highly infectious patients (Fig. 4.2).

Denyer Willis and Chandler echo this sentiment:

The saddling of responsibility for hygiene with individuals who have limited ability to change the environment in which ‘good hygiene behaviour’ is expected to operate leaves these individuals to find solutions that are more feasible and within their control, such as the use of antibiotics. (2019: 3)

Another potential solution to the individual risk the health workers face is to decide not to uncover and dress infected wounds (see below). The COM-B behaviour change model presented in the Application Guidance (Fig. 10.1007/978-3-030-62662-4_1) emphasises the importance of ‘opportunity’ to behaviour change. In our experience providing training in hand hygiene without access to resources fails to translate into improved behaviour (unsurprisingly) and acts as further demotivation as it underlines the failure of institutions to honour their duty of care to protect employees.

This chapter reports on the process of improving the IPC infrastructure. This has been achieved through continual discussion and co-decision-making. Informed by our previous experiences of formal training (Ackers et al. 2016), the team resisted the temptation to wade in and ‘train’. This does not mean that no education or knowledge creation/mobilisation took place. Rather that it evolved through team working.^8^

 Concerns about hand hygiene and the sustainability of our (previous) intervention stimulated a proposal (at the start of the project) to the hospital which would have supported the co-production and co-financing of IPC consumables and infrastructure repairs to support implementation of the hand hygiene protocol throughout the whole hospital. The proposal was based on principles of Public–Private Partnership, as envisioned by the Ministry of Health’s Strategic Plan. Unfortunately, at that point, the hospital felt unable to agree to this proposal.^9^ However, given the existence of project funding and the importance attached to reducing infection risks both for patients and health workers, the team agreed to continue providing hand gel for the ward. This has involved placing more robust dispensers on the walls in key locations (such as the sepsis area) but also, in December 2019, providing health workers with their own refillable personal dispensers. One of the intern doctors interviewed on the ward showed us his dispenser, attached to his uniform. It is interesting to see how he specifically refers to using the gel after a procedure and to protect himself:

IPC is improving - even when we cannot wash our hands, we all now have our own hand gel so after any procedure or examination we use this alcohol. It has made life safer for us. What they send through National Medical Stores is not enough – at least now we have this. This has really helped us improve patient care.

The mechanism used to improve hand hygiene on the ward has prioritised infrastructural repairs and supplies of running water, soap and hand gel. We firmly believe that Ugandan health workers are aware of the importance of hand washing both to their own well-being and that of their patients. Formal training courses (referred to locally as Continuing Medical Education or CMEs) conveying that knowledge at this stage in the project would have been inappropriate. We are also aware that health workers in Uganda (as in the UK) do not always exercise exemplary behaviour (they do not apply their knowledge to practice). In our experience, effective and continuous role modelling and mentoring in the context of good leadership is the only way to build an IPC culture. The project team included a Ugandan medical educationalist and midwife who had previously worked with us on the hand hygiene project and undergone high-level training in IPC through the Infection Control Africa Network (ICAN) programme.^10^ This midwife is Ugandan and comes from the local region so is fluent in the main local language (Rutoro). This has enabled her to build excellent relationships on the ward supporting staff with wound dressing and other duties whilst also developing a contextually appropriate version of the WHO Hand Hygiene Compliance Tool and Infrastructure Audit Tool.^11^ The in-charge nurse noted the effectiveness of this approach and, perhaps surprisingly, the fact that local staff did not feel threatened by her presence:

[K4C midwife] is on the wards at times watching them hand wash and pulling them up. Not criticising but making them constantly aware of the importance of hand washing to themselves and the patients.

One of the intern doctors noticed the improvement:

The project has really improved on IPC. These days it is a must to clean your hands and staff are using the hand sanitiser.

The following midwife echoes a concern we were familiar with from our previous project; namely the challenge of drying wet hands where there are no disposable towels^12^:

We have improved hand hygiene because we have the sinks repaired and we have enough alcohol sanitiser – there is soap and running water. Now if you don’t wash your hands that is your attitude.

[Do people wash their hands now then?]

Actually, it has changed – some do, and some feel hand washing takes time to dry but with the sanitiser you can move quickly between patients and wash hands after the procedures. There are no towels, so we use pieces of gauze.

The K4C midwife suggests that whilst much improvement has been made on hand hygiene compliance there is room for more:

I’m seeing a bit of improvement – they are using hand sanitiser – I can identify this from the WHO forms. I can identify areas they tend to forget. At least most of them remember the hand sanitiser but there is gap when they go to a new patient. Those who forget – previously they worked on patients without having it in mind to clean their hands and some of them it is still in their trait.

[So, should we put more sanitisers on the walls?]

It is working for some, but we are looking at the distance between the beds and the sanitiser and we need one fixing to the wall in the sepsis area.

The WHO Hand Hygiene Observation Tool^13^ was used to audit hand hygiene compliance. Observation took place during day shifts where most procedures are performed. The common procedures include operations such as bed-cleaning, patient examination, wound dressing and drug administration. Hand Hygiene compliance was assessed twice; first in October 2019 and secondly, in March 2020 (Table 4.2).

**Table 4.2:** Hand Hygiene Compliance in October 2019 on PNG ward at FPRRH

	Hand Washing			Hand Gel		
Cadre	Opportunities	Actual	Compliance (%)	Opportunities	Actual	Compliance (%)
Midwives	54	18	33	54	23	43
Intern Doctors	36	2	6	36	8	22
Senior Doctors	12	2	16	12	4	33

*Source* Adapted WHO Hand Hygiene Compliance Audit

The results show relatively poor adherence to WHO Hand Hygiene targets in the first observation period. We did not assess compliance prior to the project so this will represent a marked improvement on the previous period especially when sinks were not working, and hand gel was not present on the wards. It is interesting to note that compliance with hand gel use is stronger than hand washing, and midwives and nurses have higher compliance rates than doctors. Maina et al.’s study in Kenyan hospitals reported qualitative findings suggesting that, ‘nurses are more conversant with infection prevention issues’ and complaints by nurses that, ‘doctors don’t embrace the issues of IPC’ (2019: 13).

Table 4.3 shows the results for a second phase of observation in March 2020. By this point, all staff had personal hand gel bottles as well as access to dispensers at the entrance to every ward. The observer in this phase distinguished nurses and midwives (which we had not done previously) and as is common, no senior doctors were present on the ward during observations.

**Table 4.3:** Hand Hygiene Compliance in March 2020 on PNG ward at FPRRH

	Hand washing			Hand sanitiser		
Cadre	Opportunities	Actual	Compliance (%)	Opportunities	Actual	Compliance (%)
Midwives	26	20	76	26	23	88
Intern Doctors	12	7	58	12	11	91
Nurses	12	8	66	12	7	58

*Source* Adapted WHO Hand Hygiene Compliance Audit

The nurses and midwives received informal feedback after the previous observations and were aware that observation had been taking place. Table 4.3 shows marked improvement in the second observation phase especially amongst intern doctors who show a strong preference for using alcohol gel with compliance increasing from 22 to 91%, and hand washing, from 6 to 58%. Higher levels of compliance were observed among midwives than nurses. The placement of hand sanitiser on the trolleys, on walls and in the health workers’ possession have improved compliance. Observation also indicated very strong compliance with guidelines on the use of gloves (which are generally available). In both observation periods, glove utilisation was at 100%. Table 4.4 identifies compliance rates at key opportunities for hand hygiene in the second observation.

**Table 4.4:** Hand Hygiene ‘Moments’ and Compliance (March 2020) on PNG ward at FPRRH

Action/‘Moment’	Number	Percentage
Before approaching a new patient	09	45
Before aseptic action with patient	13	65
After body fluid	19	95
After completing a procedure with a patient	15	75
Total	56	

*Source* Adapted WHO Hand Hygiene Compliance Audit

**Fig. 4.3:**
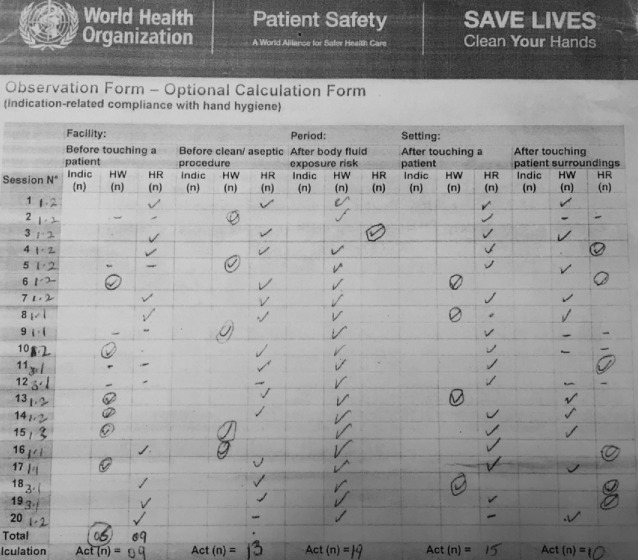
An example of the WHO Calculation Form (March 2020 Observations)

Analysis of the 56 ‘moments’ identified during this observation period showed that 95% of health workers washed hands *after* body fluid exposure and 75% remembered to wash their hands *after* touching the patient’s surrounding. Some chose to use hand gel rather than hand washing (Fig. 4.3).

Figure 4.2 gives a flavour of how the tool has worked in practice. It also shows differences in the use of hand washing and hand gel. Before touching a patient, there is a mix of staff using hand washing and hand gel whereas staff are more likely to use hand gel before commencing an aseptic procedure. Nearly all staff washed their hands after exposure to body fluids. Hand gel is much more likely to be used after touching a patient or touching patient surroundings. The results indicate a high level of compliance with IPC advice and efficient combination of the two dimensions of hand hygiene. Monistrol et al. identify hand hygiene as the ‘most important procedure’ in preventing HCAI (2011: 1212). Their intervention in a tertiary hospital in Spain, focused mainly on education and training, demonstrated marked improvements in hand hygiene compliance (also using the WHO Tool) with compliance improving from 54.3 to 75.8%. This improvement was witnessed in a facility where rooms were shared by only 2 or 3 patients and alcohol hand gel was available at every bedside. Given the far more restricted access to hand gel in the PNG, the rate of improvement is remarkable. It is interesting to note that Monistrol et al. also found poor physician compliance in comparison to nurses and, post-intervention greatest improvement amongst physicians. These findings are echoed in our study particularly with hand gel use. The use of hand gel on unsoiled hands is described by Monistrol et al. as the ‘new standard of care’; it is also far easier to implement in resource-poor environments with weak infrastructure. It is interesting to see the results of the ‘5 moments of hand hygiene’ in the Spanish study with lowest compliance ‘*before* patient contact’. The authors describe this as evidence that hand hygiene compliance is highest when health workers feel that it is protecting them (rather than the patient); ‘self-protection was the main driver for performing hand hygiene’ (p. 1217). This would explain the high rate of compliance in the moment *after* connecting with patients’ body fluids (Table 4.4).

##  IPC Infrastructure

The emphasis on identifying an intervention model for future scalability led to continuous improvement of audit tools to ensure optimal contextualisation. This meant that results are not directly comparable. The action-orientation of the MSI and our concern to develop and trial appropriate tools was more important than achieving a controlled, comparative, sample. We are also very aware that K4C presence on the wards may have contributed a ‘Hawthorn Effect’^14^ (as reported by Monistrol et al. 2011). Our concern to identify optimal methods of knowledge mobilisation, through co-presence and co-working relationships no doubt accentuates this effect. The presentation of hand hygiene audit data here is to illustrate trends; the quantitative data are heavily influenced by the intervention and we firmly believe that this type of data is best complemented by on-going observation and qualitative interviewing. Clearly the infrastructural investment made in supplying hand gel was a major driver of change. The provision of hand gel was a ‘quick fix’ preliminary intervention that stimulated an unfolding identification of other infrastructural IPC concerns.

The WASH FIT initiative is one of the World Health Organisation’s responses to critical concerns about Patient Safety.^15^ Weber et al. (2018) identify key aspects of infrastructure in LMICs that undermine progress in improving patient safety. They cite a study by Cronk and Bartram (2018) based on aggregated information from 78 LMICs which reports that 50% of health facilities studied lacked piped water; 33% did not have improved sanitation; 39% did not have soap and water for hand washing and 39% lacked proper medical waste management.

During preliminary observational phases of the MSI, we were increasingly aware of serious infrastructural challenges that would undermine our ability to bring about those behaviour changes required to reduce and manage hospital acquired infections effectively. The project team, led by the midwives on the ground, made an initial assessment of the ward infrastructure which was later followed up during a field visit by the project leads. This confirmed the need for certain investments to promote IPC on the ward, in the designated sepsis area and in a partitioned area (known as the evacuation or procedure room) used for minor procedures such as wound closing which we later identified as an intervention focal point. The team also discussed the contextual relevance of the existing WHO Infrastructural Survey Tool^16^ which colleagues felt was too focused on hand hygiene and neglected issues like signage and the status of furnishings (closing cupboards and doors etc.). We also had concerns about the section in the WHO Tool on provision of clean drinking water. This is very rarely provided in Ugandan public health facilities unless a local donor does so. We were acutely aware of the value of providing access to clean drinking water to ensure women are hydrated. However, having discussed this with local staff and the ward in-charge we made the decision not to provide clean drinking water given the serious problems associated with large numbers of visitors on the wards (see below). One of the K4C midwives reported back to the UK lead on this on-going discussion:

I talked to [the in-charge] about the issue of drinking water for patients. She did not welcome it, based on the behaviours of both patients and attendants. She felt the attendants are unruly, and she looked at the possibility of them using the drinking water for brushing and other things. She also felt sustainability may be a question. The other concern was IPC related and we should not commit ourselves by introducing drinking water on the ward.

This illustrates the ethnographic quality of the work continuing even at a distance and the value of this approach in guarding against the unintended consequences (externality effects) of seemingly easy and benevolent interventions. Some 12 months later, stimulated by ongoing concerns about the impacts of poor hydration on wound healing in several severe sepsis cases, we agreed to provide a large water boiler in the nursing station to enable staff to boil tap water and provide it to patients. This intervention may obviate the need for cannulation in cases where intra-venous fluids are given in Uganda as a substitute for oral hydration but carry their own HCAI risks (and costs).

We also had concerns about the sections of the WHO Tool auditing provision of paper towels and a waste basket for used paper towels. To our knowledge, no public health facilities in Uganda have paper towels. We therefore decided to remove these two sections of the WHO Tool from our audit. The Modified Infrastructure Audit Tool includes a list of key components with a scoring column that supports a quantitative overall score for audit and comparison purposes.^17^

## Improvements in the Infrastructure Score

A first audit, using the original WHO Tool, was completed in October 2019. This identified concerns around the sterility of equipment and instruments, ready access to hand sanitiser and healthcare waste. We also noted the absence of adequate hand-washing facilities, lack of labelling of soap bottles and display of hand hygiene posters. By this time, the project had already provided hand gel. The general patient environment was also much improved. However, we noted weaknesses under the equipment heading in terms of broken trolleys, cleaning of medical devices and re-use of single use items. Although gloves were generally available and used, there were concerns about access to eye shields and protective masks. Concerns were also expressed about the quality of waste management with health workers failing to sort waste properly and waste bins often over-loaded with poor adherence to management of sharps. These observational findings echoed Maina et al.’s conclusion that waste management is one of the weaker aspects of IPC (2019). Another continuing and persistent challenge has been in improving very poor-quality documentation and record-keeping. The Infrastructure Audit was repeated 3 times; in October 2019, January 2020 and March 2020. The overall results are presented in Table 4.5.

**Table 4.5:**
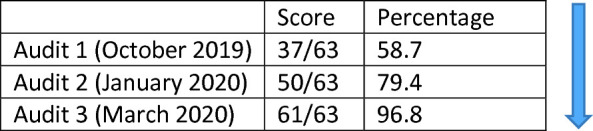
Results of IPC Infrastructure Audit

*Source* Infrastructure Audit

Audit 3 raised several residual issues (inadequate cleaning of equipment between patients; lack of labelling of waste boxes and lack of data on in-service IPC training). The following section summarises the ‘problems’ identified. Interventions took place in cycles gradually reducing gaps and improving the opportunities for effective and holistic IPC. Quite often as one problem is solved another emerges either simply because it opens a new process but also because of the kinds of externality effects referred to above.

The intervention kicked off with an early decision to re-decorate the ward. This was largely cosmetic but gave the opportunity for a thorough deep clean. The process was also designed as a motivational exercise to promote staff and patients’ sense of well-being and team working. In response to concerns about the ‘openness’ of the existing sepsis area wooden doors were fitted to isolate the sepsis area and try to prevent attendants sleeping and eating on the floor. The state of the mattresses on the ward was a great concern to heath workers as the condition enabled body fluids to enter and soak into exposed foam. The team purchased 20 new waterproof mattresses and an additional 12 waterproof covers for the mattresses that were least damaged. In response to a request from midwives and nurses, hard wearing washable aprons were provided for staff to use during wound dressing procedures to protect themselves and the patients.

**Fig. 4.4:**
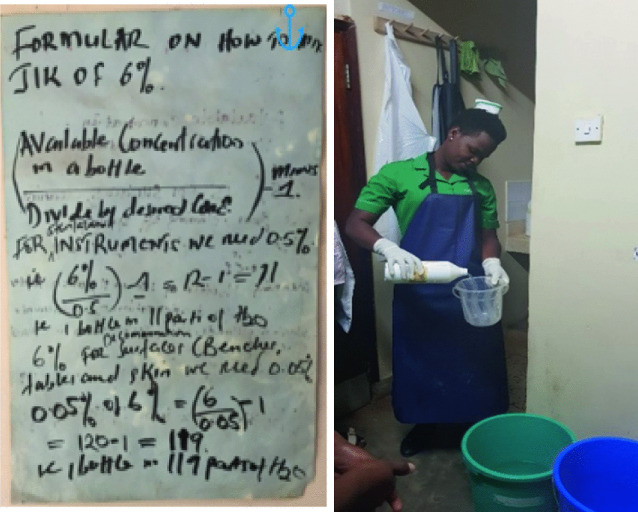
Formulae for JIC dilution

Chapter 10.1007/978-3-030-62662-4_6 discusses the problem of stock-outs in the hospital not only of drugs but also essential IPC consumables. At regular intervals, the hospital runs out of ‘JIK’, a bleach used ubiquitously in differing dilutions for many aspects of cleaning and disinfection in Ugandan hospitals. As a rule, K4C does not provide consumables to facilities as we believe this to be unsustainable. However, we did make a personal donation of 2 large bottles when the hospital ran out. One of the problems in the use of JIK was not simply its absence, but the tendency to use it far too concentrated, which damages materials and equipment. Use of JIK in this way had caused major damage to examination beds, hospital screens and instruments on the ward. This illustrates the merits of continuous observational engagement and an acute understanding of context. When we asked the hospital pharmacist about this, he reported that health workers found it hard to understand the formulae for JIC concentration on the wall in PNG (below). When we assessed this guideline, we immediately understood the major weakness in this attempt at science communication expressed as a knowledge gap on the part of nursing staff. The guideline was replaced with a simple plastic measuring jug, marked to guide the proportion of JIK to water, and buckets and training in the use of these and the problem was immediately resolved (Fig. 4.4).

Continuous engagement on the ground has enabled us to identify some of these simple problems and rectify them. The issue of dilution was picked up again once the project had purchased a new examination couch. K4C has witnessed very rapid rusting on such beds in the past where staff use neat JIK to clean metalwork. Pharmacy have provided advice on appropriate dilution of JIK for cleaning of infrastructure and furnishings and advised cleaning the bed with hand gel. Advise was also provided on the correct dilution of hydrogen peroxide which is used when dressing septic wounds. When not correctly diluted, this can cause harm by delaying healing processes. Staff are now continually trained and mentored using this guidance.

## Repair of Sinks

Staff were conscious that there were only 2 sinks on the wards and one of these, in the evacuation room where secondary closures and other procedures take place, was not functioning. Lengthy discussion took place about the pros and cons of providing an additional sink. Although this immediately seemed like a good idea to the foreign team, local staff recognised that sinks can themselves become a source of infection, especially when simultaneously used as a sluice for disposal of body fluids. On that basis, the local team decided to repair the one sink in the evacuation room and not to provide an additional sink on the main ward.

They also identified the need for new trolleys in the evacuation room and on the ward as existing trolleys failed to move (their wheels did not work) and were very old and rusted. Staff also used the same trolley for both septic and non-septic wounds (a practice which has now changed). Cupboards were also placed in the evacuation room and stainless steel (sterilisable) galley pots were provided to maintain sterility. Additional instruments were purchased so that work could continue when one set were in the process of being sterilised. At that time, there was no sterilisation equipment on the ward and the only autoclave in maternity was not working. Midwives had to walk up to the surgical ward and wait until the sterilising unit there was available wasting valuable staff time on the ward and reducing the use of the procedure room. The following excerpts indicate appreciation of interventions and the impact on behaviour:

Nowadays I’m happy because we have sterile swabs. We didn’t have instruments, so you put your hands in (the wounds). Now we have instruments we can do it properly.

[Do you feel that protects you as well?]

Yes, because nowadays we have really improved on hand washing. We have hand gel, aprons and masks. They are giving hand gel now to improve on infection because after dressing you use gel and you are free of infections. At first, we didn’t have those things.

[When you said some of the midwives didn’t want to change dressings because they were smelly, do you think they were also worried about catching infections themselves?]

Yes, because at first when you said, ‘who is going for dressing?’’ she would reply; ‘Who is going there? I am not protected.’ They were fearing infection from the patient to them. You would go there without an apron (gloves were there) but still most people would not like to go there. Now this has improved (Midwife).

A laboratory scientist echoes this view:

IPC has changed for the best. This is very positive and has been sustained through support with materials.

The following midwife notes benefits in terms of health worker safety and productivity:

The new trolleys have really helped. We can now sort our equipment out and this eases our work. The instruments in the evacuation room means we can now do procedures that used to take a long time to do as we had to sterilise equipment between cases. Mothers can be treated much more quickly, get better and leave the next day. This takes pressure off theatre too. We now have enough instruments and receivers to use (a receiver is like a kidney dish). We had very few so if we used them, we had to wait for another cycle to sterilise them before we used them again.

K4C staff expressed concern that the system of disinfecting instruments requires further improvement:

In the evacuation room some of the instruments are being left lying in jik – they should only be left in jkc for 10 minutes. We are trying to improve on it. They know they should remove the instruments from the jkc. If they put them in, by the time they have finished getting the patients ready to go back on the ward they should take them out. We had that discussion in the last staff meeting and it was agreed that if the doctors have finished the procedure, they have to make sure they alert the nurses to wash the instruments and hand over the evacuation room and then they should lock it.

This quote shows how IPC issues are raised at most (every) staff meeting, not always as a distinct training intervention but within the normal course of events. At this time, midwives had to carry instruments, gauze, etc. to surgical theatre for sterilisation. This involved staff leaving the ward and often waiting in surgical theatre for the steriliser to be available. The project has now provided a dedicated autoclave to speed this process and reduce unnecessary movement of people and instruments between wards. The discussion above illustrates the incremental and progressive approach we have taken, gradually identifying and responding to many small issues to improve both IPC and productivity. Despite the above interventions, the in-charge remained concerned at the level of over-crowding:

We are currently faced with ‘overwhelming numbers’ and yesterday there were 56 patients in a ward with a bed capacity of 40 so this is an on-going problem. This meant that there were many floor cases in the gynae area and into the sepsis zone.

This overcrowding is continuing to occur despite reduced patient stays. In many respects, it is outside of the hospital’s control as many patients continue to be referred from other health facilities including Hospitals and Health Centre IV facilities who should be able to cope with c-sections and post-natal patients. Overcrowding, combined with lack of hospital beds leading to floor cases, and inadequate sterilisation of hospital tools are factors that Denyer-Willis and Chandler (2019: 3) suggest contribute to HCAI and unnecessary prophylactic antimicrobial prescribing.

## Infection Prevention and Control and Wound Management

Flexibility and reflexivity are critical to high impact action-research. These are respected qualities in complex intervention research (Moore et al. 2015; Richards and Hallberg 2015). As noted above, this can cause some creative tension with funding bodies who, for accountability purposes, are keen to adhere as closely as possible to projected activities, associated budgets, and international protocols. The success of the MSI has derived from its very grounded, inductive, approach and the acute attentiveness to contextual dynamics. The comments of the Senior Administrator that, ‘*No little thing is ignored*’ capture this attention to context and to processes on the ground that together contribute to antimicrobial resistance and shape our ability to respond effectively to it.

Although the decision to focus on surgical site infections and take samples for laboratory testing had been planned for some time, the rationale for this focus was that many of these women would be otherwise well and that this would enable us to identify hospital acquired infections. And, although we had planned to swab c-section wounds as the basis for the laboratory testing and our AMR surveillance activity, we had not anticipated how important wound care itself was to infection control and the management of antimicrobial resistance. The opportunity to engage more actively in wound management, as a key constituent of an holistic AMR intervention, was neither mentioned in the Call Specification, in our application or indeed in the National Action Plan. It came about as a result of the recruitment of one of the first UK volunteers to the project; a nurse who had extensive experience of working on surgical site infection studies in a London hospital and a strong interest in wound management. This midwife was a ‘diaspora’ volunteer; a Ugandan national fluent in the local language (Rutoro). This, coupled with her commitment K4C’s approach to active co-working, really helped to establish rapport. On arrival, she immediately noticed patients with very badly infected, gaping wounds, and poor practices in terms of wound care. As in the case of IPC, rather than immediately commence ‘ training’ or design protocols, she worked alongside local staff to understand the context within which SSI wounds were developing and contributing to sepsis. The team spent an intensive two weeks engaging in structured observation and follow-up of 71 women who had had a c-section at FPRRH during that period. This enabled them to identify a number of concerns including; the lack of any consistent approach to the cleaning, dressing and swabbing of wounds; patient observations (essential to the early identification and management of infection and sepsis) and prophylactic antibiotic use (with prophylactic doses not completed in 94% of observed cases).

This initial observational phase also identified concerns about IPC processes (discussed above) including the use of unsterilized gauze for wound dressing; the disposal of infectious waste; the lack of sealed containers and cupboards for storage of sterilised gauze and instruments and poor management of materials on badly rusted and dysfunctional trolleys. Analysis of the 71 cases revealed a re-admission rate of 10% confirming the findings of subsequent interview data that many women were leaving the ward and returning several days later with badly infected wounds.

It was for this reason that the team embarked on intense, continuous, mentoring on wound care. One of the local midwives had already taken an active interest in wound management which she described as the most neglected area on the ward. She suggested that, when she arrived on the ward, her focus on wounds was perceived negatively:

I found the ward stinking. There was so much sepsis I went to where the smell was worst. The staff were running away from the bad smell. Some women stayed for over 2 months. There were staff shortages and many patients. I said, ‘let me look at these wounds’ so I started there. Some midwives were dressing wounds, but they were reluctant. The work was too much. It depends on someone’s interest, but it was a major priority for me. Gynae was somehow neglected; everyone was shying away because of the smell. They knew it was smelling but didn’t know what to do.

Staff were encouraged to get into the habit of documenting and reading patient notes, dressing wounds, and using simple tools developed to observe and evaluate the effectiveness of their approach. They created their own medication records following receipt of laboratory results and started a Ward Report Book. The in-charge echoes the observation discussed above:

The staff are now identifying and dressing wounds; when I arrived and before the project started the midwives didn’t do this. This resulted in a terrible smell throughout the ward which has now gone; you can smell the place is better?

She commented on the work K4C had introduced on wound cleaning:

Before that the midwives didn’t do it – midwives often focus on the pelvis^18^ and not on bedside nursing. I am a midwife and a nurse and appointed as a nurse in my role. Midwives would have been taught the theory of dressing wounds in their training but had never used those skills in practice. K4C staff really encouraged staff to start to identify wounds and treat them. The number of cases going back to theatre as a result of infected wounds has dropped significantly; they can now be better managed on the wards. This has been important in decongesting theatre and was better for the mothers. All the local staff are now engaged in wound dressing; there has been a real change in staff attitudes. Before there was no one to remind them of their knowledge and skills. They did not have the idea to manage wounds, some did not have the knowledge and there was resistance by midwives who felt it wasn’t their role. Then those of [K4C] came and this has made the job a lot more pleasant; people are enjoying work more.

It is wonderful to see not only the impact on patients’ wounds and the decongestion of the theatre but also to hear that health workers on the ward were beginning to enjoy work; this is the environment that creates meaningful opportunities for behaviour change. Another local midwife who has become actively involved in wound dressing speaks of how practice in this area has been transformed and suggests that this is also a reflection of improved IPC and provision of basic materials which protects them:

Wound dressing has totally changed. At first, we used not to dress the wounds every day for sure. Nurses didn’t want to dress the wounds because they were stinking. Sepsis had increased on the wards but nowadays staff want to do dressing because the wounds are not smelling like at first.

[Did you know how to do wound dressing?]

Yes, we were taught how to sterilise gauze and do dressings but the problem when we reach the ward you just stop because you don’t have things to use on the wards. Nowadays I’m happy because we have sterile swabs. We didn’t have instruments so you put your hands – now we have instruments – we can do it properly. At first when they said ‘who is going for dressing’ she would reply; ‘I am not protected so they were fearing infection from the patient to them so you go there without an apron – gloves were there – but still most people would not like to go there. Now this has improved because we now do dressings twice a day, they don’t get smelly.

Another local midwife, also actively involved in wound dressing on the ward, was proud to report on the case of a mother who ‘ran away’ from another health centre (Kamwenga, a 65 km distance); ‘She came here and we dressed her wound and were able to make her better’. She said that she used to dress wounds but had learnt new techniques and the importance of documentation and, when a patient appears unwell, of taking vital signs^19^:

We just tried to clean the wounds. With the guidance of the K4C colleagues we really now know that we have to take vital observations. We call them baseline bedside observations. Then if the temperature changes or the pulse we know something is happening, so we know to do vitals. We document them to find if they are stable or not. With the help of [K4C midwife] we have time. When these people are there, we can do this so right now we do. I knew wound dressing before, but I have learnt higher techniques and also recording exactly what you see on that day in the notes. Documentation is sometimes a problem, but we are trying to improve. If you come and dress the wound and don’t document no one else will know but now it helps team working. Your colleagues will also come and if you REALLY document then someone else will come – and if the wound is still bad after 3 days of wound dressing we can ask – why is this wound not getting better?

One of the intern doctors also remarked on improved wound dressing comparing practices favourably to other hospitals he had worked in:

Wound management has improved greatly. Before and in other hospitals I’ve worked in we recommend twice a day dressing and they are not changed even once. Wound management has improved greatly, and mothers are receiving twice daily dressings, so they improve so quickly. The staff have developed a **trait to inherit** – it’s a great impact.

The use of the phrase ‘trait to inherit’ would indicate a degree of continuous behaviour change or culture change in practices on the ward. The point about the smell on the ward is repeated by many respondents both in interviews and in casual conversations on the ward. Certainly, the ward no longer smells, and this has improved the working environment for all staff and patients. There has been lengthy debate on the ward about the use of honey and sugar in wound care. Whilst the use of honey is more widely accepted as having an evidence base (and antimicrobial qualities) the use of sugar, instigated by one midwife following several years of exposure to wound treatment in other settings, has received conflicting views. The midwives on the ward are clear that applying both sugar and honey to the wounds speeds healing and significantly reduces odour. Intern doctors and pharmacists are less convinced of sugar’s healing properties with one referring to its use as ‘bush medicine’. Having said that there was no suggestion that using sugar had a negative effect.^20^

This chapter has addressed the issue of infection-prevention and the contribution that quite simple and cost-effective interventions can have in reducing the incidence of infection on the wards and managing those infections that do exist more effectively. Improved IPC reduces the volume of wound infections per se. This is clear from the marked reduction in readmissions onto the ward with infected wounds. Wound management is also a dimension of IPC with an emphasis on the control aspect. And it is this attention to observing and managing wounds that created the opportunity for collection and analysis of antimicrobial resistance and, subsequently, antimicrobial use.

## Footnotes

1 https://www.who.int/gpsc/country_work/gpsc_ccisc_fact_sheet_en.pdf.

2 https://www.cdc.gov/hai/ssi/ssi.html.

3 A laparotomy is a surgical incision into the abdominal cavity, for diagnosis or in preparation for major surgery and is commonly used in obstetrics and gynaecology cases.

4 Mawalla recorded a significantly higher SSI rate amongst those men who did have surgery which they suggested may be due to multiple risk factors such as smoking and HIV infection.

5 http://www.global-pps.com/ The value of the GPPS process is discussed below.

6 Patients who come into the hospital with a pre-existing infection are regarded as having acquired that from the ‘Community’ which may imply their home environment. In many cases, these patients will have acquired an infection from a previous health facility and referred on to the hospital. In such cases CAI and HCAI are conflated and difficult to distinguish.

7 A version of this chapter has been written up as a policy document for the hospital and includes photographs of the ward and more details of the various interventions and associated costs. This can be found at www.knowledge4change.org.uk/.

8 Our approach to knowledge mobilisation in the MSI is discussed in more detail in Chapter 10.1007/978-3-030-62662-4_8.

9 We return to the PPP discussion in Chapter 10.1007/978-3-030-62662-4_9.

10 This involved a one-week training program in South Africa and another in Cameroon with an emphasis on infection prevention to improve on hand hygiene, sanitation and health care waste.

11 https://www.who.int/gpsc/5may/moment1/en/.

12 The Hand Hygiene project piloted the use of re-usable single-use hand towels. This has remained effective only in the smaller Health Centre 3 facility that K4C is partnering with. In FPRRH, the risks associated with abandoned dirty towels and the demands of apportioning staff to wash them in the absence of laundry machines outweighed the potential gains.

13 https://www.who.int/gpsc/5may/tools/en/.

14 The Hawthorne effect is a term used to describe the possibility that individuals involved in an experiment or study modify their behaviour in response to their awareness of being observed.

15 The WHO define Patient Safety as ‘the absence of preventable harm to a patient during the process of health care’, https://www.who.int/patientsafety/en/.

16 https://www.who.int/gpsc/5may/tools/evaluation_feedback/en/.

17 The team extended use of this tool across the whole hospital to support of COVID-19 intervention.

18 We assume that the reference to pelvis here is not anatomical as such but an indication that midwives focus on deliveries rather than wound management.

19 Whilst in other settings vital signs will be taken for all patients, the practice in this context is to take vital signs when a patient is showing signs of deterioration.

20 This is an ongoing debate which we will return to in due course.
